# Case report: Recurrent syncope as initial symptom in a patient with neck lymphoma

**DOI:** 10.3389/fcvm.2022.932798

**Published:** 2022-08-17

**Authors:** Yanfang Wu, Deyan Yang, Luxi Sun, Xiqi Xu, Peng Gao, Kangan Cheng, Taibo Chen, Zhongwei Cheng, Yongtai Liu, Quan Fang

**Affiliations:** Department of Cardiology, Peking Union Medical College Hospital, Chinese Academy of Medical Sciences and Peking Union Medical College, Beijing, China

**Keywords:** syncope, carotid sinus syndrome, neck malignancy, lymphoma, chemotherapy

## Abstract

Syncope may have many different causes, requiring careful identification. Recurrent syncope is uncommon as an initial symptom of neck lymphoma. Head and neck tumors involving the carotid artery cause syncope associate with carotid sinus syndrome. We report the case of a 72-year-old man who suffered from recurrent syncope due to compression of the right carotid sinus by diffuse large B-cell lymphoma and was successfully treated with immunochemotherapy. Syncope may be an early or sole sign of a neck or head tumor. We should be aware of the possibility of an underlying malignancy in patients with unexplained syncope after initial evaluation.

## Introduction

Syncope is defined as a transient loss of consciousness due to cerebral hypoperfusion, characterized by a rapid onset, short duration, and spontaneous complete recovery (1). The pathophysiological mechanism is a fall in systemic blood pressure, which is caused by low total peripheral resistance, low cardiac output, or both. For patients with recurrent syncope, diagnosis and treatment are urgently required. While etiology may be broad, syncope resulting from head and neck malignancy is rare. We describe a patient with recurrent syncope due to neck lymphoma causing compression of the carotid sinus who was successfully treated with immunochemotherapy.

## Case presentation

A 72-year-old man was admitted to our hospital with recurrent syncope over the past month. Syncope occurred while sitting, standing, or walking, with prodromes such as dizziness and palpitations. The syncope lasted 1 to 3 min. The patient developed persistent post-auricular and occipital pain in the morning. When the pain was at its peak, syncope was induced. A new, non-tender neck mass demonstrating progressive enlargement was observed. The patient had an occasional choking cough and dysphagia but no dyspnea. The patient did not complain any chest pain, exertional dyspnea or palpitation. The patient had a history of hyperthyroidism, but after that he take thyroxine tablet because of hypothyroidism. He had a history of smoking for 40 years and a family history of coronary atherosclerotic heart disease. In the physical examination, a soft mass with no tenderness was palpable in the right neck, which was misdiagnosed as an enlarged thyroid gland. Meanwhile, an enlarged lymph node (5 cm of max diameter) was palpable in the left inguinal region, with poor mobility and no tenderness. The cardiopulmonary physical examination was normal. During hospitalization, there were many episodes of syncope. Continuous tracking with a Holter monitor captured one episode of syncope, which occurred with sinus rhythm and a heart rate of 70 beats per minute. Transthoracic echocardiography showed impaired relaxation (mitral E/A ratio, 0.8) with left atrial enlargement and normal systolic function. A 2-h electroencephalogram showed that there was no epileptic seizure during the episode of syncope. Neuroimaging with computed tomography (CT) and magnetic resonance angiography were normal. Carotid artery ultrasonography revealed atherosclerotic plaques in bilateral carotid arteries with no stenosis. Considering the risk of carotid plaque embolization, carotid sinus massage was not performed. Supine and standing blood pressure measurements were not accompanied by any abnormal decrease in blood pressure. An upright tilt-table test was then performed, showing a decrease in arterial blood pressure (>20 mmHg) that reproduced syncope for a duration of 20 s without any change in heart rate, consistent with reflex syncope of the vasodepressor type.

**FIGURE 1:**
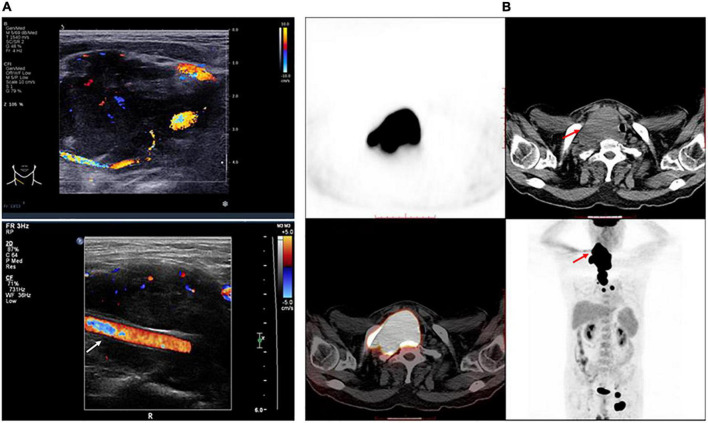
Features of the tumor. **(A)** Right neck ultrasonography shows a hypo-echoic region with abundant blood flow surrounding the right common carotid artery, which has normal internal blood flow. White arrow indicates the right common carotid artery. **(B)** The trunk positron emission tomography/computed tomography shows a large mass in the right neck surrounding the adjacent large vessels, which had a non-uniform increase in radioactive uptake. Red arrow indicates the tumor.

**FIGURE 2:**
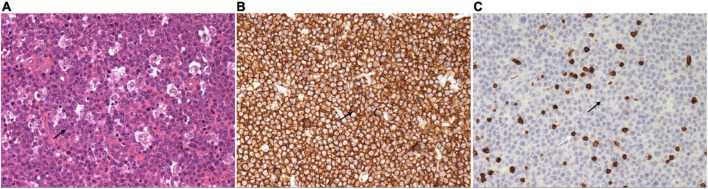
Left inguinal lymph node biopsy shows diffuse large B-cell lymphoma. **(A)** Large, round, or ovoid tumor cells are seen, with some cells having an irregularly shaped nuclear membrane. Single large or multiple small nucleoli can be seen in the tumor cells, and nuclear division is more common (H and E, × 200). **(B)** Immunohistochemistry stain of CD 20 shows the tumor cells were uniformly and strongly positive (× 200). **(C)** Immunohistochemistry stain of CD 3 shows the tumor cells are negative, but the T lymphocytes in the background are positive (× 200). Black arrow indicates the tumor cell. White arrow indicates the T lymphocyte.

Neck ultrasonography showed a hypo-echoic region with abundant blood flow in the lower middle part of the right neck, approximately 9.0 cm × 8.2 cm × 4.4 cm, surrounding the right common carotid artery, which had normal internal blood flow (Figure 1A). Positron emission tomography (PET)/CT showed a large soft tissue mass in the right neck, approximately 8.6 cm × 5.0 cm × 11.0 cm, surrounding the adjacent large vessels (Figure 1B). The mass had a non-uniform increase in radioactive uptake, with a maximum standard uptake value (SUVmax) of 59.1. Lymph nodes in the right neck (SUVmax, 30.6), in the left hilar and mediastinal regions (SUVmax, 5.3–48.6), and along the left external iliac vessels and bilateral inguinal vessels (especially left inguinal) (SUVmax 2.3–43.2) demonstrated increased metabolism. Due to the high bleeding risk of the right neck mass, the left inguinal lymph node was biopsied instead. Histopathological examination showed highly invasive B-cell lymphoma with a morphology consistent with diffuse large B-cell lymphoma (Figure 2).

**FIGURE 3:**
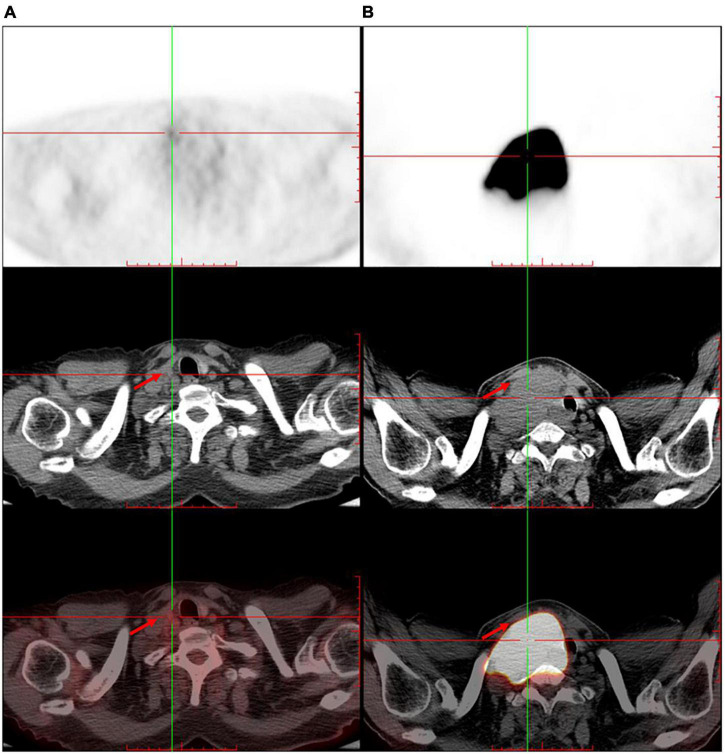
The trunk positron emission tomography/computed tomography shows significant shrinkage of the original large mass in the right neck after four cycles of immunochemotherapy. (A) After immunochemotherapy for four cycles. (B) Before the immunochemotherapy. Red arrow indicates the tumor.

The patient was referred to the hematology department for further treatment. After the assessment of the involved site, he was diagnosed as having diffuse large B-cell lymphoma (Ann Arbor stage, IV; International Prognostic index, 2) and secondary carotid sinus syndrome (CSS). He received immunochemotherapy, i.e., rituximab (600 mg), plus cyclophosphamide (1.2 g), doxorubicin (60 mg), vincristine (4 mg), and prednisone (100 mg/day × 5 day) (R-CHOP). After four cycles of R-CHOP immunochemotherapy, PET/CT revealed that the original large mass in the neck had disappeared (Figure 3). The patient no longer complained of either syncope or pain from the post-auricular and occipital regions. Choking when eating was also relieved (Table 1).

**TABLE 1:** Time line.

One month prior to presentation	• Syncope occurred while walking with prodromes such as dizziness and palpitations. Syncope occurred once a day. • cTnI: <0.1 ng/ml; BNP: 225 pg/ml. ECG: sinus rhythm and a heart rate of 65 beats per minute. Arterial blood pressure monitoring: mean blood pressure 121/76 mmHg (range: 87–161/59–98 mmHg). • Pulmonary artery computed tomography angiography: no thrombosis was observed in pulmonary artery and its branches. • Neuroimaging with CT, magnetic resonance angiography and electroencephalogram: normal. • Magnetic resonance imaging of cervical spine: hyperostosis of cervical 4–7 with narrow intervertebral space and left foramen.
Half 1 month prior to presentation	• Syncope occurred every 2–3 days. The patient developed persistent post-auricular and occipital pain in the morning. When the pain was at its peak, syncope was induced. A new, non-tender neck mass demonstrating progressive enlargement was observed. The patient had an occasional choking cough and dysphagia but no dyspnea. • Seven days-Holter monitor: long intervals and conduction block were not observed without episodes of syncope.
At presentation	Syncope occurred while sitting with prodromes such as dizziness, along with fecal incontinence. After 5–6 min, the patient regained consciousness. • cTnI: <0.017 ng/ml. ECG: sinus rhythm and a heart rate of 98 beats per minute. Continuous tracking with a Holter monitor captured one episode of syncope, which occurred with sinus rhythm and a heart rate of 70 beats per minute. Transthoracic echocardiography: impaired relaxation (mitral E/A ratio, 0.8) with left atrial enlargement and normal systolic function. • A 2-h electroencephalogram: no epileptic seizure during the episode of syncope. Carotid artery ultrasonography: atherosclerotic plaques in bilateral carotid arteries with no stenosis. • Supine and standing blood pressure measurements: normal. Upright tilt-table test: a decrease in arterial blood pressure (>20 mmHg) that reproduced syncope for a duration of 20 s without any change in heart rate. • Neck ultrasonography: a hypo-echoic region with abundant blood flow in the lower middle part of the right neck, surrounding the right common carotid artery. PET/CT: a large soft tissue mass in the right neck, surrounding the adjacent large vessels. Histopathological examination of left inguinal lymph node: diffuse large B-cell lymphoma. • Treatment: rituximab (600 mg), plus cyclophosphamide (1.2 g), doxorubicin (60 mg), vincristine (4 mg), and prednisone (100 mg/day × 5 day) (R-CHOP).
Four months later	• After four cycles of R-CHOP immunochemotherapy, PET/CT revealed that the original large mass in the neck had disappeared. • The patient no longer complained of either syncope or pain from the post-auricular and occipital regions. Choking when eating was also relieved.

cTnI, cardiac troponin I; BNP, type B natriuretic peptide; ECG, electrocardiogram; BP, blood pressure; CT, computed tomography; PCT, positron emission tomography.

## Discussion

Carotid sinus syndrome has a prevalence of 8.8% in patients aged 40 years and above with unexplained syncope after the initial evaluation (2). CSS is associated with many types of space-occupying lesions of the head and neck (3–7). Head and neck malignancy was reported as being the most frequent malignancy associated with syncope. All previously reported cases had carotid sinus compression or infiltration. Our patient, whose right carotid sinus was surrounded by neck lymphoma, presented with recurrent episodes of syncope as the initial symptom.

The pathophysiological mechanisms involved in syncope-associated CSS are complex (6). The carotid baroreceptor, located at the bifurcation of the carotid artery, is sensitive to mechanical pressure. When the arterial wall is stretched by a tumor, information is transmitted by the carotid sinus nerve to the brainstem centers in the caudal nucleus of the solitary tract. Interneurons activate the efferent pathways, resulting in the stimulation of the parasympathetic system and inhibition of the sympathetic system, which leads to a decrease in blood pressure and heart rate. Two hypotheses have been proposed to explain the association between syncope and neck lymphoma (4, 8): first, the lymphoma may invade the carotid sinus and cause direct compression, or invade the glossopharyngeal nerve, resulting in increased activity in the reflex arc; second, syncope may present as autonomic failure, similar to other B-cell lymphoma symptoms. Our patient had a large mass in the right neck surrounding the right common carotid artery that was the probable cause of syncope. The precipitating factor may be head movements. However, in clinical practice, obtaining a clear history of carotid sinus stimulation is challenging. Claassen et al. (9) reported that a turn of the head to look sideways can contribute to syncope in a healthy 70-year-old woman for the first time. In this case, we didn’t see a clear-cut relationship between head movements and symptom episodes, such as syncope caused by the head tilting to the right side, or cessation of syncope resulting from the head turning to the left side. Although a history of syncope following mechanical manipulation of the carotid sinuses is important, the absence of history does not exclude CSS.

In this case, considering the risk of carotid plaque embolization, carotid sinus massage was not performed. The current European Society of Cardiology guidelines recommend that carotid sinus massage should be avoided in patients with prior transient ischemic attack, stroke or carotid stenosis >70%, seeking to minimize neurological complications. Data was limited about the risk of neurological complications caused by carotid sinus massage in patients with carotid plaque or carotid atherosclerosis. In two prospective study with excluding patients with carotid stenosis or history of stoke, the incidence of neurological complications is low. In a prospective series of 1,000 consecutive patients aged 50 or over, only 1% had neurological symptoms during or after carotid sinus massage, such as visual disturbance, sensation of numbness, or leg weakness (10). Most complications were transient, only 0.1% patients had persistent neurological complications. Subsequently, in a prospective study with 1,401 patients (49.5% aged 80 or over), there were no neurological complications occurred during or after carotid sinus massage (11). Although the incidence of neurological complications is low, given the severity of stroke, we should inform the risk-benefit analysis when consenting patients. Therefore, carotid sinus massage should be recommended in patients with high clinical suspicion of carotid sinus syndrome after informing the risk-benefit analysis, regardless of carotid atherosclerosis, but not in patients with carotid stenosis. However, syncope associated with head and neck malignancy is often not reproducible with carotid sinus massage (7).

Additionally, our patient had persistent pain from the post-auricular region to the occipital region, which could have induced syncope. Some case reports described glossopharyngeal neuralgia, a cause of syncope in the head-and-neck tumor patient, characterized by acute unilateral head or neck pain preceding each syncopal episode (12, 13). The syncope produced by glossopharyngeal neuralgia is not completely understood. In our patient, the major mechanism may involve invasion of the glossopharyngeal nerve by malignancy (14). With glossopharyngeal afferent impulses precipitated by mechanical touch, swallowing, or taste in the posterior oropharynx, spillover impulses into the glossopharyngeal reflex arc would result in symptomatic syncope.

Management depends on the type of syncope and its cause. Syncope in patients with head and neck tumors, frequently due to vaso-depression, is under-recognized as in our case (8). Cardiac pacing rarely ameliorates these symptoms. Head and neck tumor debulking and other tumor-targeted treatments may be the best options for relieving syncope. Our patient had no further episodes of syncope after immunochemotherapy for the lymphoma. Similarly, most of the previously reported lymphoma cases also described the successful treatment of syncope after chemotherapy. Symptoms can rapidly respond to treatment of the underlying tumor. In cases with glossopharyngeal neuralgia, antiepileptic drugs or surgical resection of the glossopharyngeal nerve may be an option.

## Conclusion

In conclusion, we report a case of neck lymphoma with recurrent syncope as the initial symptom that was successfully relieved by immunochemotherapy. Syncope may be an early or sole sign of neck or head tumor. Hence, we should be aware of the possibility of an underlying malignancy in patients with unexplained syncope after initial evaluation. The diagnostic approach for syncope should include a complete medical history—particularly for tumors—and a detailed physical examination of the head and neck.

## Data availability statement

The original contributions presented in this study are included in the article/supplementary material, further inquiries can be directed to the corresponding author.

## Ethics statement

Written informed consent was obtained from the participant for the publication of this case report. Written informed consent was obtained from the individual for the publication of any potentially identifiable images or data included in this article.

## Author contributions

YW drafted the original manuscript and contributed to the case collection. DY identified by the case and revised the manuscript. LS contributed to the case collection. XX, PG, KC, TC, ZC, YL, and QF contributed to major diagnosis and treatment. All authors contributed to the article and approved the submitted version.
